# Supplementary figures and images for: Highly regenerative species-specific genes improve age-associated features in the adult Drosophila midgut
Source: BMC Biol. 2024 Aug 2;22:157. doi: 10.1186/s12915-024-01956-4 (PMC11295675; doi:10.1186/s12915-024-01956-4)

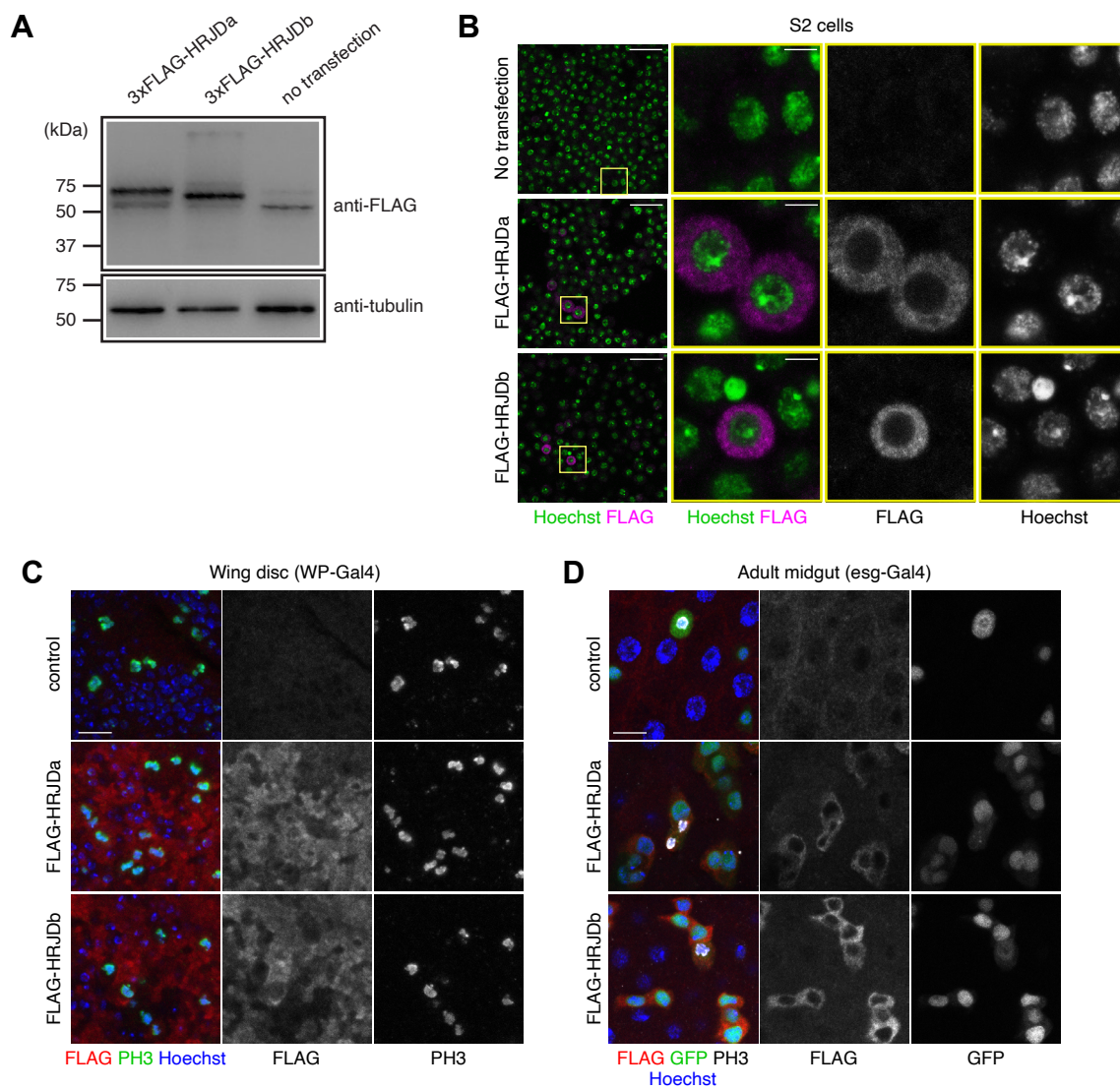

Supplement: Supplementary file 1 — Additional file 1: Figs. S1-S4. Fig. S1 - Expression and localization of HRJD proteins. Fig. S2 - Continuous expression of HRJDs throughout development hampers intestinal regeneration. Fig. S3 - Organismal lifespan of flies expressing HRJDs in ECs and ISC/EBs. Fig. S4 - HRJD induction in adult ISCs/EBs promotes turnover of midgut epithelial cells. [file 12915_2024_1956_MOESM1_ESM.zip › figure_S1.pdf]

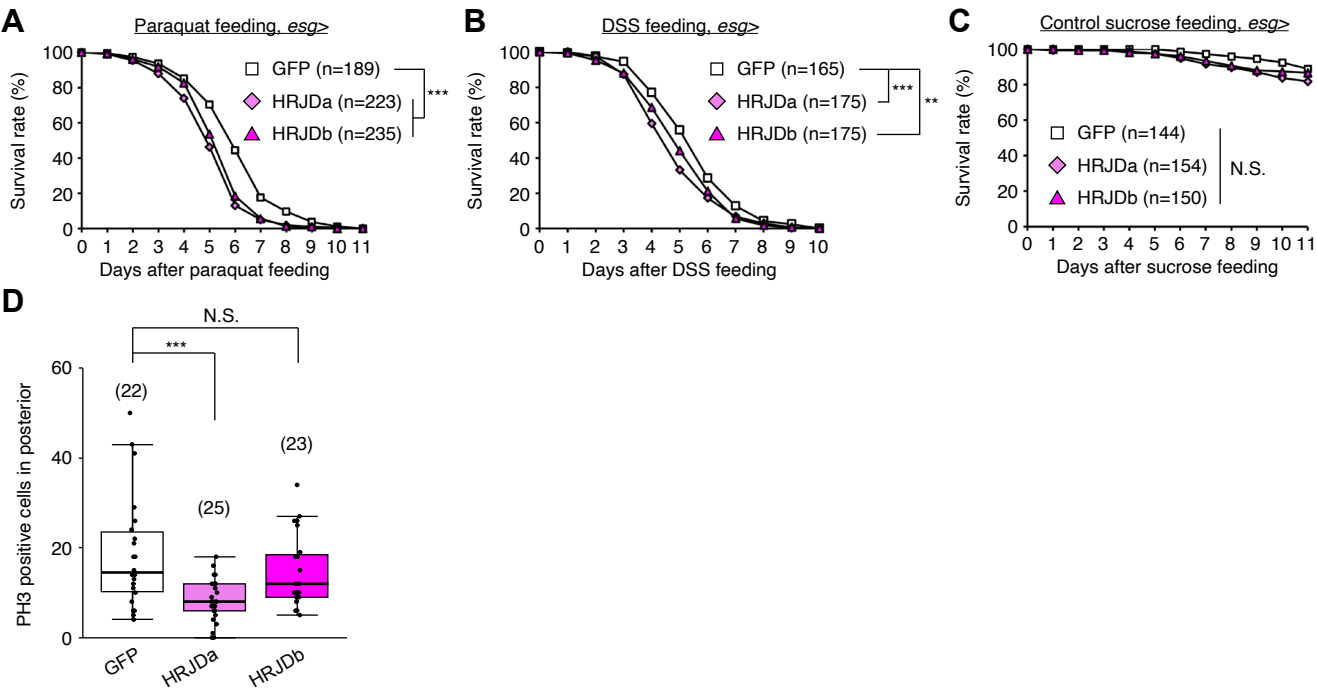

Supplement: Supplementary file 1 — Additional file 1: Figs. S1-S4. Fig. S1 - Expression and localization of HRJD proteins. Fig. S2 - Continuous expression of HRJDs throughout development hampers intestinal regeneration. Fig. S3 - Organismal lifespan of flies expressing HRJDs in ECs and ISC/EBs. Fig. S4 - HRJD induction in adult ISCs/EBs promotes turnover of midgut epithelial cells. [file 12915_2024_1956_MOESM1_ESM.zip › figure_S2.pdf]

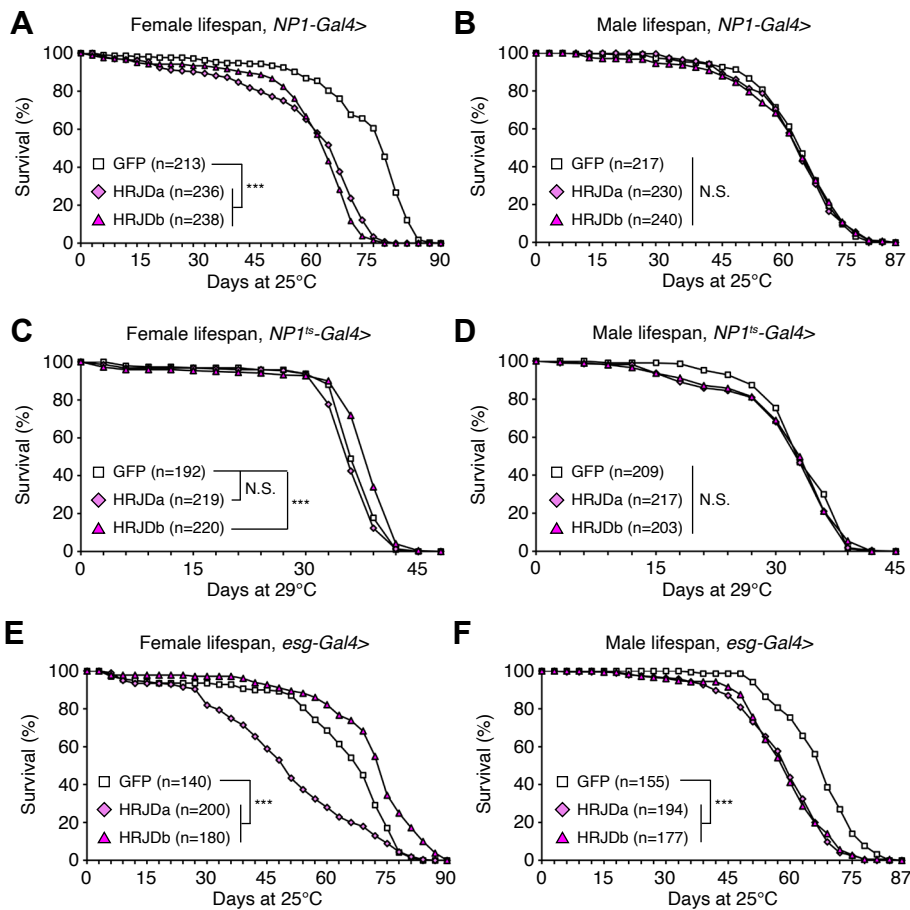

Supplement: Supplementary file 1 — Additional file 1: Figs. S1-S4. Fig. S1 - Expression and localization of HRJD proteins. Fig. S2 - Continuous expression of HRJDs throughout development hampers intestinal regeneration. Fig. S3 - Organismal lifespan of flies expressing HRJDs in ECs and ISC/EBs. Fig. S4 - HRJD induction in adult ISCs/EBs promotes turnover of midgut epithelial cells. [file 12915_2024_1956_MOESM1_ESM.zip › figure_S3.pdf]

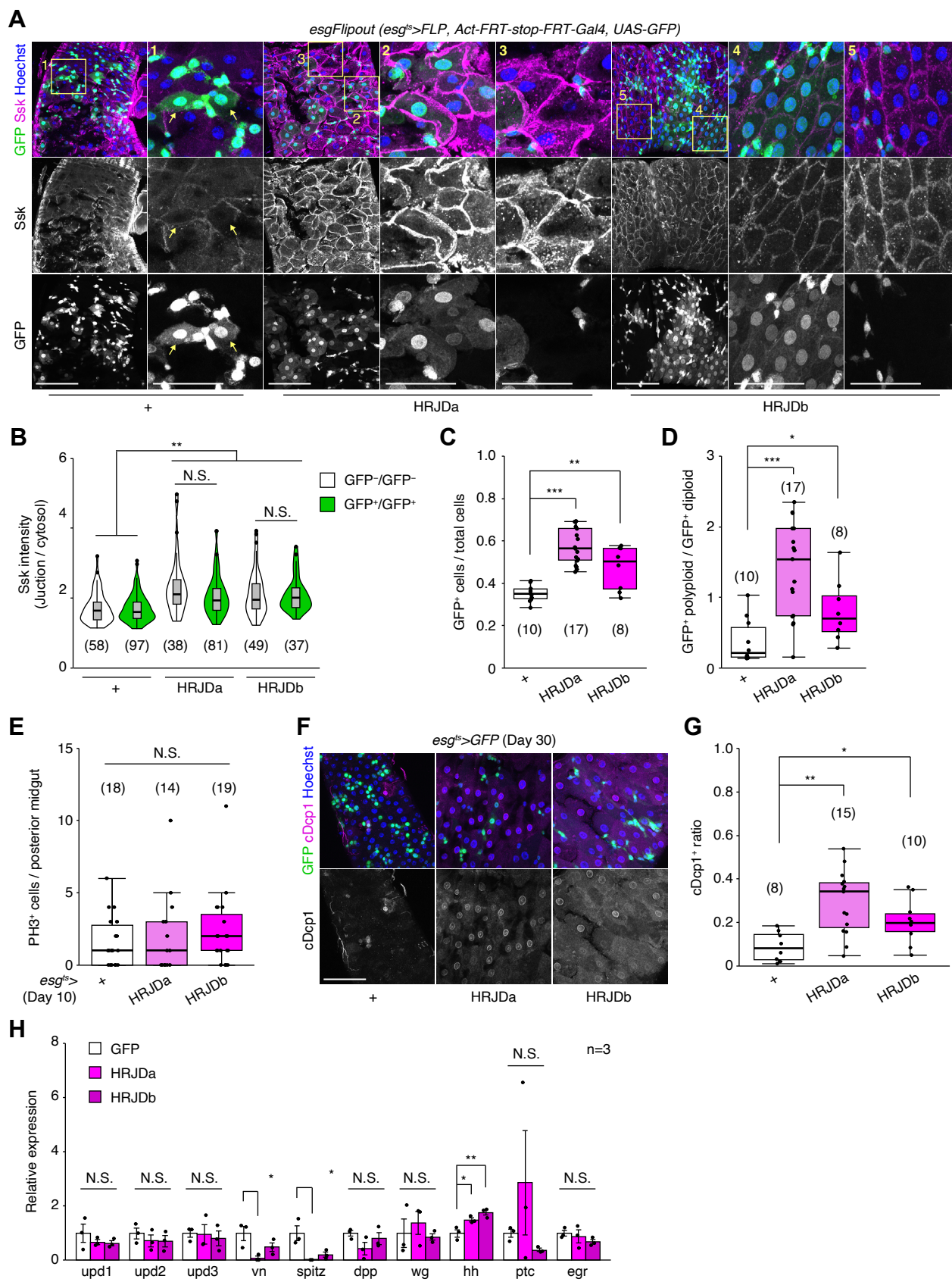

Supplement: Supplementary file 1 — Additional file 1: Figs. S1-S4. Fig. S1 - Expression and localization of HRJD proteins. Fig. S2 - Continuous expression of HRJDs throughout development hampers intestinal regeneration. Fig. S3 - Organismal lifespan of flies expressing HRJDs in ECs and ISC/EBs. Fig. S4 - HRJD induction in adult ISCs/EBs promotes turnover of midgut epithelial cells. [file 12915_2024_1956_MOESM1_ESM.zip › figure_S4.pdf]
