# Supplementary material for: Highly regenerative species-specific genes improve age-associated features in the adult Drosophila midgut
Source: BMC Biol. 2024 Aug 2;22:157. doi: 10.1186/s12915-024-01956-4 (PMC11295675; doi:10.1186/s12915-024-01956-4)
Supplement: Supplementary file 3 — Additional file 3: Supplementary texts S1. Sequences for pUCIDT-HRJDa and pUCIDT-HRJDb. [file 12915_2024_1956_MOESM3_ESM.pdf]

Supplementary texts S1

Followings are the sequences of pUCIDT-DjHRJDa/b. Yellow highlights indicate HRJDa/b coding sequences.

>DjHRJDa_mouse with pUCIDT-AMP+
TCGCGCGTTTCGGTGATGACGGTGAAAACCTCTGACACATGCAGCTCCCGGA
GACGGTCACAGCTTGTCTGTAAGCGGATGCCGGGAGCAGACAAGCCCGTCA
GGGCGCGTCAGCGGGTGTTGGCGGGTGTCGGGGCTGGCTTAACTATGCGGC
ATCAGAGCAGATTGTACTGAGAGTGCACCAAATGCGGTGTGAAATACCGCAC
AGATGCGTAAGGAGAAAATACCGCATCAGGCGCCATTCGCCATTCAGGCTGC
GCAACTGTTGGGAAGGGCGATCGGTGCGGGCCTCATCGCTATTACGCCAGCT
GGCGAAAGGGGGATGTGCTGCAAGGCGATTAAGTTGGGTAACGCCAGGGTT
TTCCCAGTCACGACGTTGTAAAACGACGGCCAGTGCAACGCGATGACGATG
GATAGCGATTCATCGATGAGCTGACCCGATCGCCGCCGCCGGAGGGTTGCGT
TTGAGACGGGCGACAGATATGTTGCAGCACTTGTCCATCCTCTTCAATATACT
GGTTTTCTCATGCCGCATTTCCGGTACCCATATCAATCACGGTAAGCCATTTGG
CACTGGACCCAGCGTATTCATCGATCACATTGATGGCTTTGTCGACCCCATCA
ATTTCTACGAAAACTTTCTTAAGAAGTCCCAACCAATTCTTATGAAGCAAGCC
GCCAAAGACTTTCCCGCCGTAAGAAATTGGGATGACGAGTACTTTTTGCAAT
TGGATTTGTCTAATTATCAGAAACACACTATAAATATTGAGAAACGCAAGAAA
GAAGATAGATCACTTCAAACCATGAAAATGAACTTCCACGAGTTTGTCCGGA
CTTACAATAAGAGCGACATATACATGGTGGACCTGATACCTACATTTTTGAATA
AAGATATAATATATCCCTGGTCACTTCAATGTGATGTTTATAGGGATAACATTTA
CCAGGTTTTGATGTGGTTCAGTTCAGGTAATACTAGTTCCGTTGTCCACACTG
ATGATATGGAGAATATCAACTGCCTCATCCGGGGAGAAAAAACCTTTGTCCTG
GTGAACCCCGACAAGCACAAGGACAAGATACCATTTAAAAGAGAAGGGGCC
TACTCAGACATAGATGTCGATTCCGTCGACTTTACCAAATATAAACAAATGGA
GTCAGTCGACTTTATTATAGCTAAAATGGTCGCTGGCGACTGCCTGTATATCCC
TAAGCTGTGGATACACCAGGTACGCAGTTACCACAGAAACATAGCCATAAAT
ATCTGGTTCAGGGTCCCTACAGACGACAGTTCCGAAATGAATGAGAGTCAAA
TGAAGATGAAGTGTGAGAAATATCCTGATAATCAGAAGAGTTTTTCTAATACT
AATTTTTTGGGTCTTAATGAACCAATTTATCAAAAGATCGATGGAGTTGGCCT
TCTGAGTAAGTTTCAAAATAGGACTGTAGAAAACCTTTTGGAGATGATACTC

GAGCTGTATAAGGAAGTCGATTCAGAGATAATAGAGAAATTTAGTAAACATAT
TGAAAGTGTGATCAAGGATATCATCGATGAAATTGACAAAAATAAGAACTCC
GTTATGGATAAGGAGGAGATAGAAACTGCTAAGAGCGAGAATCTCCATCGAA
TAAGCAACTACATCAACTACTTGAAAACCTTCCTGGAGACTCTTTCCATTAAG
GACATCATAAGTCTCGAGAACAAGAAGACTATCGAATTTCACAAGTATGAGC
TCTAAATCAGTTCTGGACCAGCGAGCTGTGCTGCGACTCGTGGCGTAATCAT
GGTCATAGCTGTTTCCTGTGTGAAATTGTTATCCGCTCACAATTCCACACAAC
ATACGAGCCGGAAGCATAAAGTGTAAAGCCTGGGGTGCCTAATGAGTGAGCT
AACTCACATTAATTGCGTTGCGCTCACTGCCCGCTTTCCAGTCGGGAAACCT
GTCGTGCCAGCTGCATTAATGAATCGGCCAACGCGCGGGGAGAGGCGGTTTG
CGTATTGGGCGCTCTTCCGCTTCCTCGCTCACTGACTCGCTGCGCTCGGTCGT
TCGGCTGCGGCGAGCGGTATCAGCTCACTCAAAGGCGGTAATACGGTTATCC
ACAGAATCAGGGGATAACGCAGGAAAGAACATGTGAGCAAAAGGCCAGCA
AAAGGCCAGGAACCGTAAAAAGGCCGCGTTGCTGGCGTTTTTCCATAGGCTC
CGCCCCCCTGACGAGCATCACAAAAATCGACGCTCAAGTCAGAGGTGGCGA
AACCCGACAGGACTATAAAGATACCAGGCGTTTCCCCCTGGAAGCTCCCTCG
TGCGCTCTCCTGTTCCGACCCTGTCGCTTACCGGATACCTGTCCGCCTTTCTC
CCTTCGGGAAGCGTGGCGCTTTCTCATAGCTCACGCTGTAGGTATCTCAGTTC
GGTGTAGGTCGTTCGCTCCAAGCTGGGCTGTGTGCACGAACCCCCCGTTCAG
CCCGACCGCTGCGCCTTATCCGGTAACTATCGTCTTGAGTCCAACCCGGTAAG
ACACGACTTATCGCCACTGGCAGCAGCCACTGGTAACAGGATTAGCAGAGCG
AGGTATGTAGGCGGTGCTACAGAGTTCTTGAAGTGGTGGCCTAACTACGGCT
ACACTAGAAGAACAGTATTTGGTATCTGCGCTCTGCTGAAGCCAGTTACCTTC
GGAAAAAGAGTTGGTAGCTCTTGATCCGGCAAACAAACCACCGCTGGTAGC
GGTGGTTTTTTTGTTTGCAAGCAGCAGATTACGCGCAGAAAAAAAGGATCTC
AAGAAGATCCTTTGATCTTTTCTACGGGGTCTGACGCTCAGTGGAACGAAAA
CTCACGTTAAGGGATTTTGGTCATGAGATTATCAAAAAGGATCTTCACCTAGA
TCCTTTTAAATTAAAAATGAAGTTTTAAATCAATCTAAAGTATATATGAGTAAA
CTTGGTCTGACAGTTACCAATGCTTAATCAGTGAGGCACCTATCTCAGCGATC
TGTCTATTTCGTTCATCCATAGTTGCCTGACTCCCCGTCGTGTAGATAACTACG
ATACGGGAGGGCTTACCATCTGGCCCCAGTGCTGCAATGATACCGCGAGACC
CACGCTCACCGGCTCCAGATTTATCAGCAATAAACCAGCCAGCCGGAAGGGC
CGAGCGCAGAAGTGGTCCTGCAACTTTATCCGCCTCCATCCAGTCTATTAATT

GTTGCCGGGAAGCTAGAGTAAGTAGTTCGCCAGTTAATAGTTTGCGCAACGT
TGTTGCCATTGCTACAGGCATCGTGGTGTCACGCTCGTCGTTTGGTATGGCTT
CATTCAGCTCCGGTTCCCAACGATCAAGGCGAGTTACATGATCCCCCATGTTG
TGCAAAAAAGCGGTTAGCTCCTTCGGTCCTCCGATCGTTGTCAGAAGTAAGT
TGGCCGCAGTGTTATCACTCATGGTTATGGCAGCACTGCATAATTCTCTTACTG
TCATGCCATCCGTAAGATGCTTTTCTGTGACTGGTGAGTACTCAACCAAGTCA
TTCTGAGAATAGTGTATGCGGCGACCGAGTTGCTCTTGCCCGGCGTCAATAC
GGGATAATACCGCGCCACATAGCAGAACTTTAAAAGTGCTCATCATTGGAAA
ACGTTCTTCGGGGCGAAAACTCTCAAGGATCTTACCGCTGTTGAGATCCAGT
TCGATGTAACCCACTCGTGCACCCAACTGATCTTCAGCATCTTTTACTTTCAC
CAGCGTTTCTGGGTGAGCAAAAACAGGAAGGCAAAATGCCGCAAAAAAGG
GAATAAGGGCGACACGGAAATGTTGAATACTCATACTCTACCTTTTTCAATAT
TATTGAAGCATTTATCAGGGTTATTGTCTCATGAGCGGATACATATTTGAATGT
ATTTAGAAAAATAAACAAATAGGGGTTCCGCGCACATTTCCCCGAAAAGTGC
CACCTGACGTCTAAGAAACCATTATTATCATGACATTAACCTATAAAAATAGG
CGTATCACGAGGCCCTTTCGTC

>DjHRJDb_mouse with pUCIDT-AMP+
TCGCGCGTTTCGGTGATGACGGTGAAAACCTCTGACACATGCAGCTCCCGGA
GACGGTCACAGCTTGTCTGTAAGCGGATGCCGGGAGCAGACAAGCCCGTCA
GGGCGCGTCAGCGGGTGTTGGCGGGTGTCGGGGCTGGCTTAACTATGCGGC
ATCAGAGCAGATTGTACTGAGAGTGCACCAAATGCGGTGTGAAATACCGCAC
AGATGCGTAAGGAGAAAATACCGCATCAGGCGCCATTCGCCATTCAGGCTGC
GCAACTGTTGGGAAGGGCGATCGGTGCGGGCCTCATCGCTATTACGCCAGCT
GGCGAAAGGGGGATGTGCTGCAAGGCGATTAAGTTGGGTAACGCCAGGGTT
TTCCCAGTCACGACGTTGTAAAACGACGGCCAGTGCAACGCGATGACGATG
GATAGCGATTCATCGATGAGCTGACCCGATCGCCGCCGCCGGAGGGTTGCGT
TTGAGACGGGCGACAGATATGAATTTTATTAGTTATTTCTGTCAGTTGCTCTGT
ATCTTCAAGTGCTTCTTCCAGATCAATACAATTGAAGAGGGAGACAAAACTG
ACCTCTACTTCGTCAAGGATGATGGCATGAATGCTAAGTTCCCCCCTAAGATT
ACCAGGTCTATTCATGACCAAATACCAGTGGGCCATCTGCGGTCTCTTGGCTT
CCAGAGGATAGCAGAAGGTCCAGTAGACACAATGAAGACCTTCTTGCAACC
AGAAAACTTTTTCACCAAATATGTTCTCAAGAATAACCCCCTGGTCGTGAGA

AACGCCATTGATGAAAAGCAACCTATTAGGGTCACCTGGAATGATGAGCGCC
TTAAGAAGATACATGGGAAGAGCAAGGTTTGGGTCATCAAAAGACACGAGA
AGTCTTCCAAGGCACCAACCAAGGTTACACTGGAACGGTTTTTGGAGTTTTA
CAAGCTGCAGGACATTTACCTCGCTTCAACAATACCCGAAGAGTTTATGTCTG
AGATCACCTACCCCAAATTTATGAAGTGCGGGCCAATAGTTAACTCCCTCGTA
GAGGCAGAGCTTTGGATGGCTTACGGGGGAACCGGGTCATTGTTGCACTACC
ACGGGGACCATCAGATCCATTGCATGGCAGATGGTCGAAAGGACTTCATTCT
CATGGAGAATAAGCACAAGAAGAGCTTCAAGATGGTGGAAAAGCATCCCTT
CACAGGAGAGGGATATAGCGAACTTGATACTGAGATGGTGAATATGTTTCAG
GAAAAACAAATCTCCGGGATTCCATGGATATATGCCACCATTCTGGCTGGCGA
TTGTCTCTTCATTCCTGCAGGATACATTCACCAGGTGCGCAGCTATGGGCGGA
GCCTTAGCTACACTATTCAGTTTGCTTCAACTCATAACAAGGCTACTACTAAA
TGCGATAACCTCAGTGAAAATGACAACGATGATACCCTTATGAATCACGACTT
TATCTACACCGAACGAGATGGTATCGAGCAACTGAAGAATACAAAATTGACA
GATGAGGATCTGAGGAATATACTGATAAAACTCATCCGGACCGCCACCATATT
GCCCTTCGAGAGATTTCAGTATTTCTACAATATTGCTATTGGAAAGGTAAAAC
AGAAGATGCCAAGTGCTCAAGTGGTATTTAATAACATTGACCTGAAGCGCGA
CGGCTTCATTACACTGGAGCAAATTCAAAAACTGCCCGGTAATCAACTCAAC
AAAATCTCAAAAATACTCAATTCTATACATAAGAGTCGACACGACGAACTCTA
AATCAGTTCTGGACCAGCGAGCTGTGCTGCGACTCGTGGCGTAATCATGGTC
ATAGCTGTTTCCTGTGTGAAATTGTTATCCGCTCACAATTCCACACAACATAC
GAGCCGGAAGCATAAAGTGTAAAGCCTGGGGTGCCTAATGAGTGAGCTAACT
CACATTAATTGCGTTGCGCTCACTGCCCGCTTTCCAGTCGGGAAACCTGTCGT
GCCAGCTGCATTAATGAATCGGCCAACGCGCGGGGAGAGGCGGTTTGCGTAT
TGGGCGCTCTTCCGCTTCCTCGCTCACTGACTCGCTGCGCTCGGTCGTTCGG
CTGCGGCGAGCGGTATCAGCTCACTCAAAGGCGGTAATACGGTTATCCACAG
AATCAGGGGATAACGCAGGAAAGAACATGTGAGCAAAAGGCCAGCAAAAG
GCCAGGAACCGTAAAAAGGCCGCGTTGCTGGCGTTTTTCCATAGGCTCCGCC
CCCCTGACGAGCATCACAAAAATCGACGCTCAAGTCAGAGGTGGCGAAACC
CGACAGGACTATAAAGATACCAGGCGTTTCCCCCTGGAAGCTCCCTCGTGCG
CTCTCCTGTTCCGACCCTGTCGCTTACCGGATACCTGTCCGCCTTTCTCCCTTC
GGGAAGCGTGGCGCTTTCTCATAGCTCACGCTGTAGGTATCTCAGTTCGGTGT
AGGTCGTTCGCTCCAAGCTGGGCTGTGTGCACGAACCCCCCGTTCAGCCCGA

CCGCTGCGCCTTATCCGGTAACTATCGTCTTGAGTCCAACCCGGTAAGACACG
ACTTATCGCCACTGGCAGCAGCCACTGGTAACAGGATTAGCAGAGCGAGGTA
TGTAGGCGGTGCTACAGAGTTCTTGAAGTGGTGGCCTAACTACGGCTACACT
AGAAGAACAGTATTTGGTATCTGCGCTCTGCTGAAGCCAGTTACCTTCGGAA
AAAGAGTTGGTAGCTCTTGATCCGGCAAACAAACCACCGCTGGTAGCGGTG
GTTTTTTTGTTTGCAAGCAGCAGATTACGCGCAGAAAAAAAGGATCTCAAGA
AGATCCTTTGATCTTTTCTACGGGGTCTGACGCTCAGTGGAACGAAAACTCA
CGTTAAGGGATTTTGGTCATGAGATTATCAAAAAGGATCTTCACCTAGATCCT
TTTAAATTAAAAATGAAGTTTTAAATCAATCTAAAGTATATATGAGTAAACTTG
GTCTGACAGTTACCAATGCTTAATCAGTGAGGCACCTATCTCAGCGATCTGTC
TATTTCGTTCATCCATAGTTGCCTGACTCCCCGTCGTGTAGATAACTACGATAC
GGGAGGGCTTACCATCTGGCCCCAGTGCTGCAATGATACCGCGAGACCCACG
CTCACCGGCTCCAGATTTATCAGCAATAAACCAGCCAGCCGGAAGGGCCGAG
CGCAGAAGTGGTCCTGCAACTTTATCCGCCTCCATCCAGTCTATTAATTGTTG
CCGGGAAGCTAGAGTAAGTAGTTCGCCAGTTAATAGTTTGCGCAACGTTGTT
GCCATTGCTACAGGCATCGTGGTGTCACGCTCGTCGTTTGGTATGGCTTCATT
CAGCTCCGGTTCCCAACGATCAAGGCGAGTTACATGATCCCCCATGTTGTGC
AAAAAAGCGGTTAGCTCCTTCGGTCCTCCGATCGTTGTCAGAAGTAAGTTGG
CCGCAGTGTTATCACTCATGGTTATGGCAGCACTGCATAATTCTCTTACTGTCA
TGCCATCCGTAAGATGCTTTTCTGTGACTGGTGAGTACTCAACCAAGTCATTC
TGAGAATAGTGTATGCGGCGACCGAGTTGCTCTTGCCCGGCGTCAATACGGG
ATAATACCGCGCCACATAGCAGAACTTTAAAAGTGCTCATCATTGGAAAACGT
TCTTCGGGGCGAAAACTCTCAAGGATCTTACCGCTGTTGAGATCCAGTTCGA
TGTAACCCACTCGTGCACCCAACTGATCTTCAGCATCTTTTACTTTCACCAGC
GTTTCTGGGTGAGCAAAAACAGGAAGGCAAAATGCCGCAAAAAAGGGAATA
AGGGCGACACGGAAATGTTGAATACTCATACTCTACCTTTTTCAATATTATTGA
AGCATTTATCAGGGTTATTGTCTCATGAGCGGATACATATTTGAATGTATTTAG
AAAAATAAACAAATAGGGGTTCCGCGCACATTTCCCCGAAAAGTGCCACCTG
ACGTCTAAGAAACCATTATTATCATGACATTAACCTATAAAAATAGGCGTATCA
CGAGGCCCTTTCGTC
